# Utility of the Drain Fluid-to-Serum Creatinine Ratio for Diagnosing Postoperative Complications Following Genitourinary Surgery in Moroccan Patients

**DOI:** 10.7759/cureus.99025

**Published:** 2025-12-12

**Authors:** Kadija Mefire, Walid Salama, Samira El Machtani Idrissi, Sanae Bouhsain, Abdellah Dami, Mohammed Alami, Asmaa Biaz

**Affiliations:** 1 Biochemistry-Toxicology Laboratory, Mohamed V Military Teaching Hospital, Faculty of Medicine and Pharmacy, Mohammed V University, Rabat, MAR; 2 Urology, Mohamed V Military Teaching Hospital, Faculty of Medicine and Pharmacy, Mohammed V University, Rabat, MAR

**Keywords:** dcr/pcr ratio, drain fluid creatinine, genitourinary surgery, plasma creatinine, urinary leak

## Abstract

Background: Postoperative complications represent a major challenge in genitourinary surgery. The drain fluid-to-plasma (serum) creatinine (DCr/PCr) ratio has emerged as a simple, cost-effective biochemical marker for detecting postoperative urinary leakage.

Objective: To evaluate the diagnostic performance of the DCr/PCr ratio in identifying postoperative urinary leakage in a Moroccan patient cohort.

Methods: A retrospective study was conducted on 118 patients who underwent genitourinary surgery between May 2022 and April 2024. Simultaneous serum and drain fluid creatinine measurements, performed during the first postoperative days, were analyzed alongside clinical and demographic data.

Results: The cohort had a mean age of 65.36 years and was predominantly male (95.8%). Laparoscopic radical prostatectomy (51.7%) and radical cystectomy with urinary diversion (22.9%) were the most common procedures. The mean DCr/PCr ratio was markedly higher in patients with urinary leakage (22.48) than in those without leakage (1.63). ROC curve analysis demonstrated excellent diagnostic accuracy, with an area under the curve (AUC) of 0.977 (95% CI: 0.949-0.996). A cutoff value of ≥4.0 yielded a sensitivity of 86.7%, specificity of 97.1%, and a negative predictive value of 98.0%.

Conclusion: The DCr/PCr ratio is a highly accurate, rapid, and cost-effective tool for detecting postoperative urinary leakage. A cutoff of ≥4.0 provides excellent diagnostic performance, particularly for ruling out leakage. Routine integration of this biochemical marker into postoperative assessment protocols may improve early detection and reduce unnecessary imaging, although prospective multicenter validation remains warranted.

## Introduction

Genitourinary surgeries are commonly associated with postoperative complications, among which urinary leakage is particularly prevalent [1]. The systematic placement of surgical drains serves as a key preventive measure; however, the interpretation of drain output requires reliable diagnostic tools to identify complications promptly [2]. The biochemical analysis of drain fluid, specifically the measurement of creatinine levels, has emerged as a valuable method for detecting postoperative complications involving the kidney, bladder, or prostate, especially urinary leakage [2].

In standard practice, drain fluid creatinine is monitored serially during the postoperative period to guide clinical decisions [3]. The removal of the drain is typically contingent upon the normalization of these levels [1]. The drain fluid-to-plasma (serum) creatinine (DCr/PCr) has been identified as an effective preliminary test for screening and identifying patients with postoperative urinary leakage [3,4].

While traditional diagnosis often relies on clinical signs confirmed by radiological examinations [5], these methods may not always allow for the earliest possible detection. Early identification of urological complications, particularly anastomotic leakage, is crucial due to their substantial impact on patient morbidity, healthcare costs, and overall clinical outcomes.

Therefore, we conducted this retrospective study to evaluate the utility of the DCr/PCr ratio as an early and reliable indicator of urinary leakage in patients following genitourinary surgery.

## Materials and methods

Study criteria

We conducted a retrospective study over a two-year period, from May 1, 2022, to April 30, 2024, involving patients who underwent genitourinary surgery at the Urology Department of the Mohammed V Military Teaching Hospital (HMIMV) in Rabat. The study included 118 patients who received simultaneous postoperative measurements of plasma and drain fluid creatinine performed at the Biochemistry and Toxicology Laboratory of the same institution. Patients who underwent similar surgical procedures but did not have paired creatinine measurements were excluded.

In our clinical practice, postoperative evaluation followed a stepwise approach combining clinical, biochemical, and radiological assessment. Clinically, Redon drain output was monitored daily during the first postoperative days, with attention to the persistence or abnormal increase in clear drainage. Biochemically, creatinine measurement in the drain fluid was performed to differentiate urinary leakage from lymphatic drainage; a urinary origin was suspected when drain creatinine exceeded plasma creatinine (DCr/PCr ratio >1). Radiologically, an abdominopelvic CT scan was performed when clinical evolution or biochemical findings suggested urinary leakage, in order to confirm and localize the extravasation.

Data collection

Data were extracted from patient medical records and the Laboratory Information System (LIS). A standardized data collection form was used to record demographic characteristics, indications for surgery, types of surgical procedures performed, biochemical results, and postoperative complications for each patient.

Creatinine measurement

Surgical drain and blood samples were collected in dry or heparinized tubes and immediately transported to the biochemistry laboratory for analysis. Creatinine concentrations in plasma and drain fluid were measured using the Alinity c ABBOTT® analyzer (Abbott Laboratories, Abbott Park, IL, USA), employing the kinetic alkaline picrate (modified Jaffé) method.

This technique is based on the reaction of creatinine with picrate in an alkaline medium to form a creatinine-picrate complex. The resulting increase in absorbance at 500 nm is directly proportional to the creatinine concentration in the sample.

Statistical analysis

Statistical analyses were performed using SPSS software (version 23.0; SPSS Inc., Chicago, IL, USA). Quantitative variables were expressed as mean ± standard deviation, and qualitative variables as frequencies and percentages. Group comparisons were conducted using the Student's t-test for normally distributed variables and the Mann-Whitney U test for variables with non-normal distributions. Categorical variables were compared using the Chi-square test. A p-value < 0.05 was considered statistically significant.

The diagnostic performance of the drain fluid-to-plasma creatinine ratio (DCr/PCr) for detecting anastomotic urinary leakage was assessed using receiver operating characteristic (ROC) curve analysis. The area under the curve (AUC) and its 95% confidence interval were calculated. The optimal cutoff value was determined using the Youden index. Sensitivity, specificity, positive predictive value (PPV), and negative predictive value (NPV) were then computed.

## Results

A total of 118 patients underwent genitourinary surgery at HMIMV Rabat between May 2022 and April 2024. The cohort included 113 men (95.8%) and five women (4.2%), with a mean age of 65.36 years (range 27-89) (Table 1). The most common procedure was laparoscopic radical prostatectomy (51.7%, n = 61), followed by radical cystectomy with urinary diversion (22.9%, n = 27), both frequently associated with lymph node dissection. While radical prostatectomies were performed laparoscopically, radical cystectomies and all other procedures were carried out using open or minimally invasive approaches (Table 1).

**Table 1:** Patient characteristics, surgical indications, and procedures (n = 118). *Other indications included: ureteropelvic junction obstruction, left testicular tumor, abdominopelvic mass, intervesicorectal gastrointestinal stromal tumor, bladder stone, ileal conduit stenosis, benign prostatic hyperplasia, and acute urinary retention. **Other procedures included: adenomectomy, orchidectomy, abdominopelvic mass resection, intervesicorectal gastrointestinal stromal tumor removal, bladder stone extraction, ileal conduit revision, transurethral resection of the prostate, ureteral recanalization, and management of acute urinary retention.

Variable/indication/procedure	n (%)/Mean ± SD
Age (years), mean ± SD	65.36 ± 10.25
Sex	
Male	113 (95.8%)
Female	5 (4.2%)
Indications for surgery	
Prostate adenocarcinoma	61 (51.7%)
Bladder tumor	30 (25.4%)
Renal tumor	15 (12.7%)
Renal mass	2 (1.7%)
Post-surgical peritonitis	2 (1.7%)
Other indications (each n = 1)*	9 (7.6%)
Type of surgery	
Laparoscopic radical prostatectomy	61 (51.7%)
Radical cystectomy	27 (22.9%)
Left partial nephrectomy	9 (7.6%)
Right extended total nephrectomy	4 (3.4%)
Left extended total nephrectomy	3 (2.5%)
Anterior pelvectomy	3 (2.5%)
Exploratory laparotomy + lavage	2 (1.7%)
Other procedures (each n = 1)**	9 (7.6%)

Postoperative complications occurred in 74 patients (62.7%), while 44 patients (37.3%) had an uncomplicated postoperative course. Urinary incontinence was the most common complication (31.4%, n = 37), followed by anastomotic urinary leakage (12.7%, n = 15). Less frequent events included febrile syndrome (4.2%), wound infection (2.5%), peritonitis (2.5%), general deterioration (1.7%), intestinal obstruction (1.7%), and urinary infection (1.7%) (Table 2).

All urinary leakage cases occurred in the early postoperative period. Suspicion was raised based on persistent drainage and confirmed by biochemical analysis within the first postoperative days; CT imaging was performed when needed.

**Table 2:** Types of postoperative complications observed. *Each occurred once and includes: evisceration, chronic nephropathy, lymph node metastases, hydropneumothorax, and residual prostate-specific antigen (PSAt).

Type of complication	Frequency (n)	Frequency (%)
No complication	44	37.29
Urinary incontinence	37	31.36
Anastomotic urinary leakage	15	12.71
Febrile syndrome	5	4.24
Wound infection	3	2.54
Peritonitis	3	2.54
General health deterioration	2	1.69
Intestinal obstruction	2	1.69
Urinary infection	2	1.69
Other complications (each n = 1)*	5	4.24
Total	118	100

Patients who developed postoperative complications showed markedly higher biochemical values compared to those without complications (Table 3). Mean drain fluid creatinine was significantly elevated in the complication group (69.78 ± 165.97 mg/L vs. 9.23 ± 5.28 mg/L; p = 0.002), as was mean plasma creatinine (13.96 ± 16.91 mg/L vs. 9.68 ± 3.56 mg/L; p = 0.039). The DCr/PCr ratio was also significantly higher in patients with complications (6.26 ± 15.96 vs. 0.95 ± 0.37; p = 0.006). These findings confirm a strong biochemical distinction between complicated and uncomplicated postoperative courses.

**Table 3:** Comparison of creatinine levels and DCr/PCr ratio according to complication status. DCr/PCr: drain fluid-to-plasma (serum) creatinine.

Variable	No complication (n = 44)	Complication present (n = 74)	t-statistic	p-value
Drain creatinine (mg/L), mean ± SD	9.23 ± 5.28	69.78 ± 165.97	-3.136	0.002
Plasma creatinine (mg/L), mean ± SD	9.68 ± 3.56	13.96 ± 16.91	-2.100	0.039
DCr/PCr ratio, mean ± SD	0.95 ± 0.37	6.26 ± 15.96	-2.861	0.006

**Figure 1:**
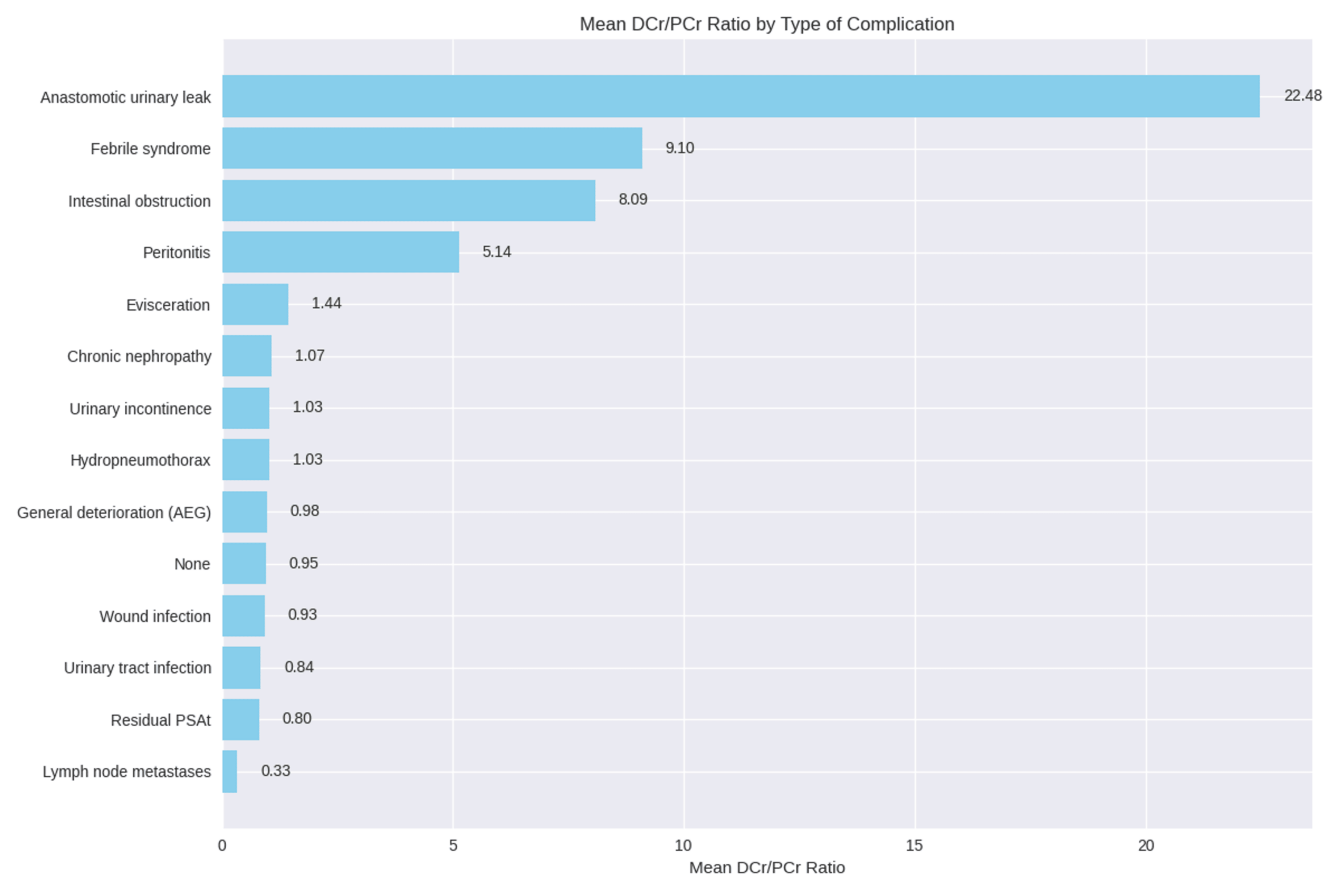
Distribution of DCr/PCr ratio according to complication type. DCr/PCr: drain fluid-to-plasma (serum) creatinine; residual PSAt: prostate-specific antigen.

As illustrated in Figure 1, the DCr/PCr ratio varied dramatically based on the complication type. Patients with anastomotic urinary leaks exhibited a markedly high mean ratio (22.48). In contrast, those with other complications, such as urinary incontinence, had a ratio near plasma levels (1.44), whereas patients without complications had a mean ratio of 0.95.

As shown in Figure 1, the mean DCr/PCr ratio varied markedly across postoperative complications. The highest ratio was observed in anastomotic urinary leaks (22.48), followed by febrile syndrome (9.10) and intestinal obstruction (8.09). Peritonitis showed a moderate increase (5.14). All other complications, including urinary incontinence, chronic nephropathy, wound infection, urinary tract infection, hydropneumothorax, and general deterioration, had ratios close to 1.0, similar to patients without complications (0.95). Lymph node metastases exhibited the lowest ratio (0.33).

The mean DCr/PCr was significantly higher in patients with anastomotic urinary leak (22.48 ± 28.99) compared to those without leak (1.63 ± 4.35), with a highly significant difference (p < 0.001) (Table 4).

The mean DCr/PCr ratio was markedly higher in patients with anastomotic urinary leakage compared to those without leakage (22.48 ± 28.99 vs. 1.63 ± 4.35) (Table 4). This difference was statistically significant (p = 0.015), confirming a strong distinction between leak and no-leak groups.

**Table 4:** Comparison of DCr/PCr ratio between patients with and without anastomotic urinary leak. DCr/PCr: drain fluid-to-plasma (serum) creatinine.

Variable	Leak (n = 15)	No leak (n = 103)	t-statistic	p-value
DCr/PCr ratio, mean ± SD	22.48 ± 28.99	1.63 ± 4.35	2.779	0.015

**Figure 2:**
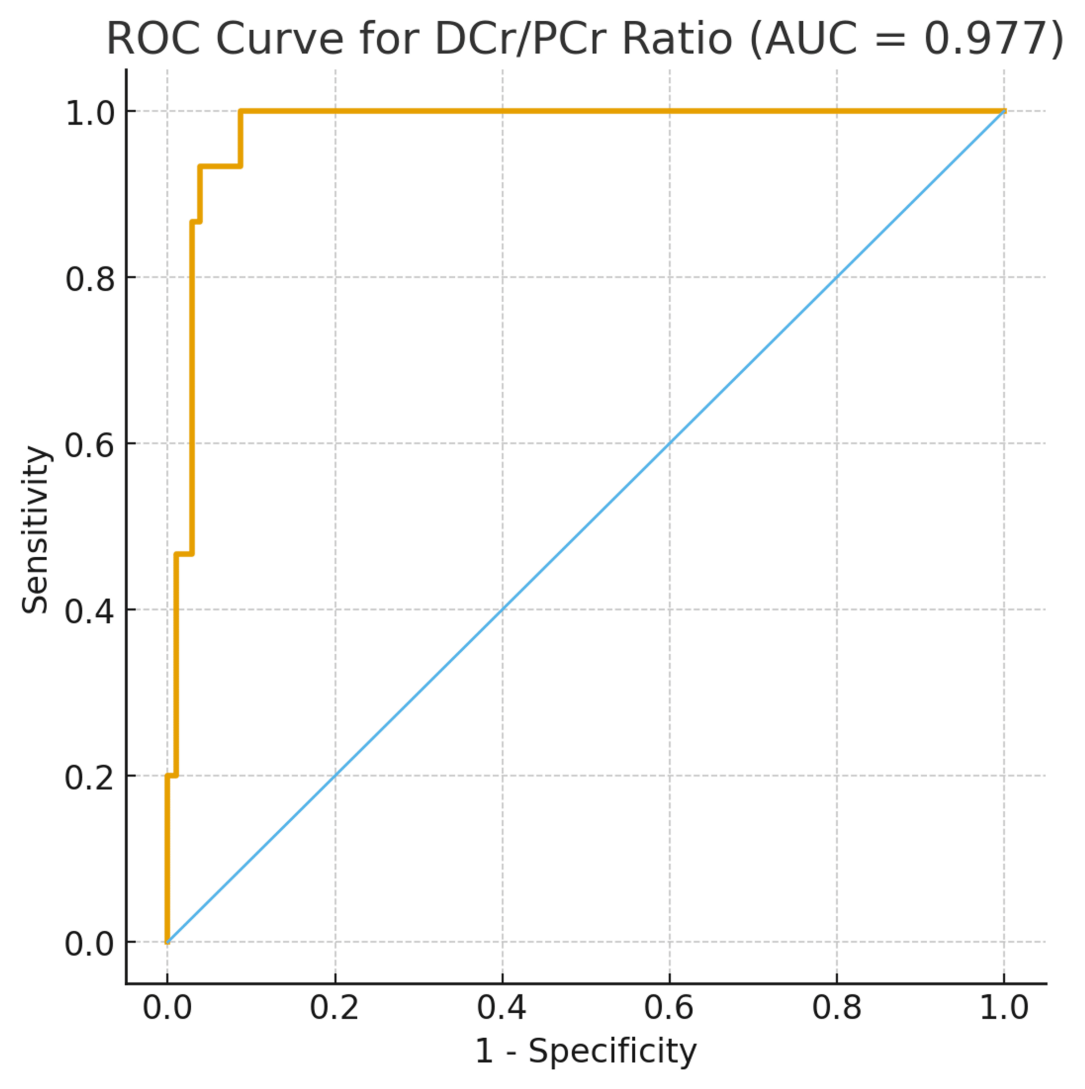
Receiver operating characteristic (ROC) curve for model performance evaluation. DCr/PCr: drain fluid-to-plasma (serum) creatinine; AUC: area under the curve.

The diagnostic performance of the DCr/PCr ratio for detecting anastomotic urinary leakage was evaluated using receiver operating characteristic (ROC) curve analysis (Figure 2). The area under the curve (AUC) was 0.977 (95% CI: 0.949-0.996), indicating excellent diagnostic accuracy. An optimal DCr/PCr ratio cutoff of ≥4.0 yielded a sensitivity of 86.7%, a specificity of 97.1%, a positive predictive value of 81.3%, and a negative predictive value of 98.0%.

## Discussion

Biochemical analysis of drainage fluid has emerged as a valuable tool for diagnosing postoperative complications of the urological tract [2]. In this context, we conducted a two-year retrospective study to evaluate the clinical usefulness of the drain fluid-to-plasma creatinine ratio (DCr/PCr) in predicting urinary complications after genitourinary surgery.

In our cohort, the mean patient age was 65.36 years, with a clear male predominance, reflecting the demographic profile typically reported in urological surgical populations. The main surgical indications were prostate adenocarcinoma (51.7%) and bladder tumors (25.7%), corresponding primarily to laparoscopic radical prostatectomy (51.7%) and radical cystectomy with urinary diversion (22.9%). The discrepancy between bladder tumor indications and cystectomy procedures is explained by the fact that some bladder tumors were treated with pelvectomy rather than cystectomy. These findings are consistent with published literature, which reports increasing prostate cancer prevalence with advancing age [6].

Several studies have suggested that very high postoperative drainage volumes may indicate urinary leakage. In particular, clear peritoneal drainage exceeding 500 mL/day has been proposed as a clinical warning sign [7], and a drain creatinine concentration only 18% higher than serum creatinine may already suggest urinary leakage [8]. In our series, patients with postoperative complications exhibited markedly elevated drain fluid creatinine levels compared with those without complications, and this elevation consistently exceeded serum creatinine levels, supporting the diagnostic utility of biochemical drain assessment.

The DCr/PCr ratio has been proposed as a simple and relevant tool to differentiate urinary leakage from other postoperative complications [8]. A classical threshold of DCr/PCr >2 is often used to suspect leakage [4,8]. In our cohort, patients with confirmed anastomotic urinary leakage showed a markedly elevated mean ratio (22.48 ± 28.99) compared with those without leakage (1.63 ± 4.35), a statistically significant difference. Extreme values--up to 89.4--corresponded to massive leakage.

Diagnostic performance analysis confirmed the strong discriminatory power of the ratio. The ROC curve demonstrated outstanding accuracy (AUC = 0.977, 95% CI: 0.949-0.996). Using the Youden index, we identified an optimal cutoff of DCr/PCr ≥4.0, yielding a sensitivity of 86.7%, specificity of 97.1%, PPV of 81.3%, and NPV of 98.0%. The extremely high NPV indicates that values below this threshold reliably exclude urinary leakage.

Our findings align with previous studies while refining the diagnostic threshold. Flores-Gama et al. reported that a ratio >6 beyond the first postoperative week increased the probability of leakage sixfold [9]. Although their cutoff was higher, our results suggest that ≥4.0 may serve as a more sensitive early indicator. Prospective multicenter studies remain needed to validate and standardize this threshold.

Measuring the DCr/PCr ratio provides a simple, rapid, cost-effective, and non-invasive screening tool for postoperative urinary leakage, allowing early detection with minimal resource use. Prior studies have similarly demonstrated the value of biochemical evaluation as an initial step preceding imaging [7-9]. Our results reinforce the importance of integrating biochemical testing into routine postoperative monitoring to reduce unnecessary imaging while maintaining diagnostic accuracy.

Consistent with recent literature reporting that complications of urogenital surgery are predominantly urinary or hemorrhagic in nature [10,11], our study found urinary incontinence (31.4%) and urinary leakage (12.7%) to be the most frequent postoperative complications, particularly following major surgeries such as prostatectomy and cystectomy.

However, interpretation of these findings must consider several limitations. The retrospective, single-center study design may introduce selection bias and limit generalizability. Additionally, the study did not compare outcomes between open and minimally invasive techniques. Variations in surgical approach and postoperative care across institutions further emphasize the need for multicenter prospective studies to validate our findings and standardize the use of the DCr/PCr ≥4.0 cutoff in clinical practice.

## Conclusions

The drain fluid-to-plasma creatinine ratio (DCr/PCr) emerges as a highly valuable and practical biomarker for the early detection of postoperative urinary leakage in genitourinary surgery. Its ability to rapidly and accurately distinguish urinary leakage from other postoperative fluid collections provides a major clinical advantage, helping prevent severe complications, optimize patient outcomes, and reduce healthcare costs by limiting unnecessary imaging. Routine integration of the DCr/PCr ratio into postoperative monitoring is strongly recommended. Automated calculation and reporting through Laboratory Information Systems (LIS) would further streamline its use, enabling earlier recognition of complications and more efficient patient management.

Our study provides robust evidence supporting the diagnostic relevance of the DCr/PCr ratio and identifies a clinically actionable cutoff value of ≥4.0, which demonstrated excellent sensitivity, specificity, and a very high negative predictive value. These results reinforce the suitability of this ratio as a standard component of postoperative assessment. Nevertheless, prospective multicenter studies are needed to validate and standardize its application across diverse clinical settings.

## References

[1] Kim JH (2011). Book review: Complications of urologic surgery: prevention and management, 4th ed. Int Neurourol J.

[2] Schmeusser BN, Nicaise EH, Palacios AR (2024). A practical approach for drain fluid analysis following genitourinary surgery. Surg Oncol Insight.

[3] Williams RD, Snowden C, Thiel DD (2017). Assessment of perioperative variables that predict the need for surgical drains following robotic partial nephrectomy utilizing quantitative drain creatinine analysis. J Laparoendosc Adv Surg Tech A.

[4] Erlich T, Abu-Ghanem Y, Ramon J, Mor Y, Rosenzweig B, Dotan Z (2017). Postoperative urinary leakage following partial nephrectomy for renal mass: risk factors and a proposed algorithm for diagnosis and management. Scand J Surg.

[5] Kulkarni JN (2011). Perioperative morbidity of radical cystectomy: a review. Indian J Urol.

[6] Rakauskas A, Marra G, Heidegger I (2021). Focal therapy for prostate cancer: complications and their treatment. Front Surg.

[7] Wang JH, Kung YH, King TM, Chang MC, Hsu CW (2015). Measurement of peritoneal fluid urea nitrogen and creatinine levels is useful to detect iatrogenic urinary tract leakage in colorectal surgery. J Chin Med Assoc.

[8] Regmi SK, Bearrick EN, Hannah PT, Sathianathen N, Kalapara A, Konety BR (2021). Drain fluid creatinine-to-serum creatinine ratio as an initial test to detect urine leakage following cystectomy: a retrospective study. Indian J Urol.

[9] Flores-Gama F, Bochicchio-Riccardelli T, Mondragón-Ramírez G (2010). Determination of creatinine in drained liquid: urinary leak or lymphocele?. Cir Cir.

[10] Mathieu R, Doizi S, Bensalah K (2022). Complications in urological surgery: prostate surgery. Prog Urol.

[11] Irani J, Legeais D, Madec FX (2022). Complications in urological surgery. Prevention. Prog Urol.

